# P53, MAPK, topoisomerase II alpha and Ki67 immunohistochemical expression and KRAS/BRAF mutation in ovarian serous carcinomas

**DOI:** 10.1186/1746-1596-8-21

**Published:** 2013-02-06

**Authors:** Dinka Sundov, Ana Caric, Ivana Mrklic, Dijana Gugic, Vesna Capkun, Irena Drmic Hofman, Branka Petric Mise, Snjezana Tomic

**Affiliations:** 1 Department of Pathology, Forensic Medicine and Cytology, Clinical Hospital Center Split, School of Medicine, University of Split, Split, Croatia; 2 Department of Radiology, Clinical Hospital Center Split, School of Medicine, University of Split, Split, Croatia; 3 Department of Nuclear Medicine, Clinical Hospital Center Split, School of Medicine, University of Split, Split, Croatia; 4 Department of Oncology, Clinical Hospital Center Split, School of Medicine, University of Split, Split, Croatia

**Keywords:** Ovary, Serous Carcinomas, Carcinogenesis, Type I, Type II, Ovar, seröse Karzinome, Karzinogenesis, Typ I Tumoren, Typ II Tumoren

## Abstract

**Background:**

We investigated the immunohistochemical expression of p53, MAPK, topoisomerase II alpha (topoII alpha) and Ki67 in ovarian serous carcinomas (OSCs) along with mutational analysis for KRAS and BRAF.

**Methods:**

Eighty one cases of OSCs were reviewed and examined immunohistochemically using antibodies against p53, MAPK, topoII alpha and Ki67. Staining was evaluated as a percentage of immunopositive cells with cut-off levels at 10% for p53 and topoII alpha, and 5% for MAPK. The Ki67 immunoexpression was assessed by means of Olympus Image Analysis System as a percentage of immunopositive cells in 1000 tumor cells. KRAS and BRAF mutational analysis was performed on 73 available microdissected samples.

**Results:**

Of 81 cases of OSCs 13.6% were of low-grade and 86.4% were of high-grade morphology. In the high-grade group there was a significantly higher immunoexpression of p53 (*P* < 0.001) and topoII alpha (*P* = 0.001), with Ki67 median 56.5 vs. 19 in low-grade group (*P* < 0.001). The difference in immunoexpression of active MAPK between low- and high-grade group was also significant (*P* = 0.003). MAPK positive immunostaining was detected in 63.6% of low-grade vs. 17.1% of high-grade OSCs. The frequency of KRAS mutation was significantly higher in low-grade as compared to high-grade group (*P* = 0.006). None of the samples had BRAF mutation. In addition, we detected positive MAPK immunoexpression in 13/59 samples with wild-type KRAS, suggesting that activation of MAPK pathway is not ultimately related either to KRAS or BRAF mutation. Seven morphologically high-grade samples (11.7%) showed both KRAS mutation and p53 immunopositivity.

**Conclusions:**

Although this study is limited by its humble number of low-grade samples, our data fit the proposed dualistic pathway of ovarian carcinogenesis. Mutational analysis for KRAS and BRAF discloses some possible interactions between different tumorigenic pathways of low- and high-grade carcinomas. Immunohistochemical staining for MAPK was not sufficiently sensitive, nor specific, to precisely predict the KRAS mutation. However, it appears to be quite reliable in ruling out a KRAS mutation if the staining is negative.

**Virtual Slides:**

The virtual slide(s) for this article can be found here: http://www.diagnosticpathology.diagnomx.eu/vs/9283563368804632

**Zusammenfassung:**

**Hintergrund:**

Wir untersuchten die Immunohistochemische Expression der p53, MAPK, topoisomerase II alpha (topoII alpha) und Ki67 in Ovarialkarzinomen (OSCs) anbei mit Mutationsanalyse für KRAS und BRAF.

**Methode:**

81 OSCs Fälle wurden analysiert und Immunohistochemisch untersucht mit Antikörper gegen p53, MAPK, topoII alpha und Ki67. Die Färbung war ausgewertet als der Prozent von immunopositiven Zellen mit den “cut-of” Niveau an 10% für p53 und topoII alpha und 5% für MAPK. Die Ki67 Expression war bewertet mittels Olympus Image Analysis System als der Prozent von immunopositiven Zellen in 1000 Tumorzellen. KRAS and BRAF Mutationsanalyse wurde in 73 verfügbaren microdissections Stichproben aufgeführt.

**Ergebnisse:**

Von 81 OSCs Fälle 13.6% zeigte “low-grade” und 86.4% “high-grade” Morphologie. In der “high-grade” Gruppe war eine statistisch bedeutende höhere Expression von p53 (P < 0.001) und topoII alpha (P = 0.001) mit Ki67 median von 56.5 im Gegensatz zu 19 in der “low-grade” Gruppe (P < 0.001). Die Differenz in Immunoexpression von aktiver MAPK zwischen der “low-grade” und “high-grade” Gruppe war statistisch bedeutend (P = 0.003). MAPK positive Expression war in 63.6% der “low-grade” im Gegensatz von 17.1% der “high-grade” Karzinoms bemerkt. Die Häufigkeit der KRAS Mutation war bedeutend höher in “low-grade” im Verglich zu der “high-grade” Gruppe (P = 0.006). Keiner der Stichproben hate BRAF Mutation. Wir haben auch eine positive MAPK Expression in 13/59 der Stichproben mit “wild-type” KRAS bemerkt, was sugeriert das die Aktivation des MAPK Pfads ist nicht letztmalig mit KRAS oder BRAF verbunden. Sieben der “high-grade” Stichproben (11.7%) waren KRAS Mutation und p53 Expression positive.

**Schlussworte:**

Obwohl diese Studie mit bescheiden Nummer von “low-grade” Stichproben limitiert ist, unsere Daten passen in das dualistische Modell von Ovarial Karzinogenesis. Mutationsanalyse für KRAS und BRAF enthüllen einige mögliche Interaktionen zwischen verschieden tumorigenen Wege von “low”- and “high-grade” Karcinomen.

Die Immunohistochemische Expression für MAPK war nicht empfindlich oder spezifisch genüg um den KRAS mutations Status des Tumor genau vorauszusagen.

Es scheint das die MAPK Expression ziemlich verlässlich ist in ausschließen der KRAS Mutation, wenn die Expression negative ist.

## Background

In the Western hemisphere ovarian carcinomas still remain the most frequent cause of death due to gynecological cancer [1]. Efforts in early detection and new insights in therapeutic approaches demonstrated no clear benefit. In a sense, we are forced to go “back to basics”.

Historically, the principal means of classifying ovarian carcinomas has been histological assessment of cell type. This approach is reflected in the current World Health Organization’s ovarian carcinoma classification [2]. Meanwhile, morphological studies upgraded by molecular genetic studies have enabled new insights into the pathogenesis of ovarian cancer with possible consequences on future cancer screening and platinum-based treatments. It has become apparent that the different subtypes of ovarian carcinoma represent distinct disease entities.

The discovery of biological differences between low-grade and high-grade serous carcinomas has provided a basis for Baltimore group led by Robert J. Kurman to propose a new dualistic model of ovarian carcinogenesis that recognized “type I” and “type II” pathways, with serous type ovarian carcinoma as a prototype [1,3].

According to proposed model, ovarian serous carcinomas (OSCs) which evolve along type I pathway are relatively indolent low-grade neoplasms that arise in a stepwise fashion from well-characterized precursor lesions and usually present as large FIGO stage I neoplasms. They often harbor somatic mutations of genes encoding protein kinases, including KRAS and BRAF, the upstream regulators of mitogen-activated protein kinase (MAPK) [4]. According to Siedman et al. [5] low-grade serous carcinomas are significantly less common than high-grade and represent approximately 10% of serous carcinomas.

In contrast, OSCs which evolve along type II pathway are aggressive high-grade neoplasms, with a larger volume of tumor occurring outside the ovaries. More than 75% of high-grade carcinomas harbor TP53 mutations. Recent data suggest that these neoplasms arise from intraepithelial carcinomas, the majority of which have been detected in the tubal fimbriae [1,6].

The loss of wild type p53 as a transcriptional suppressor may lead to unregulated or inappropriate expression of topoisomerase II alpha (topoII alpha), resulting in increased cell proliferation [7]. TopoII alpha is an enzyme with an important role in DNA topology, repair and replication, coded by a single copy gene on the locus q21of chromosome 17 [8,9]. It is a cell-cycle-related protein, expressed in normal as well as neoplastic cells in the S, G2 and M phase [8,10,11].

The immunoexpression of Ki67 antigen has become a useful tool to determine the proliferative potential of a tumor. Its high expression has been found to indicate a poor prognosis in several cancers, including ovarian [12]. The gene for Ki67 protein is located on chromosome 10q25. Ki67 protein expression is strictly connected with cellular cycle. This antigen appears in G1, S, G2 and M cellular cycle phases, remaining in hide in G0 and early G1 phase [13].

So far, it is not clear whether some high-grade serous carcinomas develop from low-grade tumors that follow type I pathway. Dualistic model implies that the pathogenesis of low- and high-grade carcinomas is separate and independent. Nevertheless, according to Dehari et al. [14] there can be rare intersections between these tumorigenic pathways.

The aim of this study was to better define ovarian serous carcinomas and their relation to type I and type II pathways, by comparing the p53, MAPK, topoII alpha, and Ki67 immunohistochemical expression in low- and high-grade morphological group along with mutational analysis for KRAS and BRAF.

## Methods

Tumor samples were obtained from the primary surgery material prior to chemotherapy. Formalin-fixed, paraffin-embedded tumor tissue samples of 81 OSCs were retrieved from the archives of the Department of Pathology, Clinical Hospital Center Split and classified as low-grade or high-grade serous carcinomas according to criteria proposed by Kurman and Shih [3].

Low-grade group corresponds to invasive low-grade serous carcinomas, mostly characterized by micropapillary and cribriform patterns, with small solid nests and cords of relatively uniform cell population with small, rounded nuclei (the degree of nuclear atypia qualifies as grade 1). Mitotic activity is low. Psammoma bodies are often present and there is no evidence of necrosis.

High-grade group corresponds to the usual type of serous carcinoma with complex papillary and solid patterns, and marked cytological atypia. Tumor cells have large, pleomorphic nuclei, and many cells are multinucleated (nuclear atypia grades 2 and 3). There is a high level of mitotic activity, and abnormal mitotic figures are frequent. Necrosis is a common feature [3,15,16].

All patients were staged according to the criteria of the International Federation of Gynecology and Obstetrics (FIGO) staging system [17].

Ethical commitee for biomedical research of the Clinical Hospital Center Split and School of Medicine approved that this research are in compliance with the Helsinki Declaration (reference number 49-1/06).

### Immunohistochemistry

The evaluation of the immunohistochemical staining was performed independently by two authors with special interest in gynecological pathology.

All procedures were performed according to the manufacturers’ protocols, using the standard streptavidin-biotin-peroxidase technique.

Paraffin 3–5 μm thick tissue sections were deparaffinized in xylene and rehydrated in descending concentrations of alcohol. To facilitate antigen retrieval, slides were treated in a microwave oven at 750 W and 110°C, 3 times for 5 minutes in a citrate buffer. Immunostainings for p53, topoII alpha and Ki67 (clone MIB-1) were performed with monoclonal antibodies to human p53 (DAKO, Glostrup, Denmark, mouse anti-human M7001, at a dilution of 1:50), topoII alpha (DAKO, Glostrup, Denmark, mouse anti-human 7816, at a dilution of 1:75) and Ki67 (DAKO, Glostrup, Denmark, mouse anti-human M7240, at a dilution of 1:200). Immunostaining for MAPK was performed with rabbit polyclonal antibody, pTEpY, which specifically reacts with phosphorylated (active) MAPK (Promega, Madison, WI, V8031, at a dilution of 1:500). All slides were incubated with labeled streptavidin-biotin followed by diaminobenzidin chromogen (DAKO). Mayer′s hematoxylin was used for counterstaining.

Nuclear staining for p53, topoII alpha and Ki67 was considered as a positive result. Positive reaction for MAPK was defined as discrete localization of the brown chromogen in the nucleus or cytoplasm. Negative controls were created by omission of the primary antibody.

Staining was evaluated according to the number of cells showing positivity (as a percentage of positive cells), within representative areas of the tumor sample. For statistical analysis, based on reports in the published literature, cut-off levels were stratified at 10% for p53 [18] and topoII alpha [9] and 5% for MAPK [19].

The Ki67 immunoexpression was assessed by means of Olympus Image Analysis System as a percentage of immunopositive cells in 1000 tumor cells.

### Mutational analysis

Paraffin blocks from 73 cases were available for molecular analysis. Genomic DNA was isolated using a High Pure PCR Template extraction kit (Roche Applied Science, Germany), according to the manufacturer’s protocol.

### PCR analysis and determination of KRAS and BRAF mutations

KRAS mutation detection in exon 1 codons 12 and 13 was performed using LightMix® Kit k-ras Mutations Codons 12/13 (Roche Diagnostics, Germany) and LightCycler® FastStart DNA Master HybProbe kit (Roche Diagnostics, Germany). PCR was performed with LightCycler 2.0 instrument (Roche Diagnostics, Germany), according to manufacturer’s recommendations.

Primers used for BRAF PCR amplification and PCR conditions were those given in Powell et al. [20] PCR was performed with GeneAmp PCR System 9700 (Applied Biosystems, Foster City, CA).

### Statistical analysis

Statistical analysis was carried out using the SPSS version 10.0 software package. The categorical variables were compared using *χ*^2^ test. Continuous variables were compared using the Mann–Whitney *U* test. *P* values ≤ 0.05 were considered statistically significant.

## Results

A total of 81 OSCs were included in this study. According to previously described morphological criteria, 13.6% (11/81) serous carcinomas in our study were low-grade OSCs and 86.4% (70/81) were high-grade OSCs.

Patients’ age ranged from 44–71 years in low-grade (median, 52) and 37–89 (median, 63.5) years in high-grade group. At diagnosis, 72.7% of patients in low-grade and 47.1% of patients in high-grade group were under the age of 60. There was no association between the tumor group and the age of patient (*χ*^2^ = 1.5; *P* = 0.194).

Seven of eleven (63.6%) patients in the low-grade group and 64/70 (91.5%) patients in the high-grade group had advanced stage disease (stages III or IV). Therefore, 35.4% of the low-grade and merely 8.6% of the high-grade carcinomas are discovered in the early FIGO stages (*χ*^2^ = 4.5; *P* = 0.026).

After surgery, 63.6% patients from the low-grade group and only 17.1% patients from the high-grade group were without residual tumor. Residual tumor larger than 2 cm was still present in 62.9% of patients with high-grade OSC, and 27.3% of patients with low-grade OSC (*χ*^2^ = 9.9; *P* = 0.019). The presence of immeasurable lesion (i.e. ascites) without solitary residual tumor was detected in 28.6% of patients in the low-grade group, and 16.7% of patients in the high-grade group.

Mitotic activity was determined as mitotic count on 10 high power fields (HPFs). Thirty-two percent of low-grade carcinomas had ≤ 2 mitoses/10 HPFs. Median in the low-grade group was 9 mitoses/10 HPFs (range, 1–12). In the high-grade group, grade 2 nuclear atypia was found in 31%, and grade 3 nuclear atypia in 69% of carcinomas. Median in the high-grade group was 27 mitoses/10 HPFs (range, 13–65). Vascular invasion was present in 71.4% of the high-grade and in only 9.1% of the low-grade carcinomas (*χ*^2^ = 13.3; *P* < 0.001).

Clinicopathological features are summarized in Table 1.

**Table 1:** Clinicopathological features of patients with OSCs

	**Low-grade**	**High-grade**	***P***
	**(n = 11)**	**(n = 70)**	
Age at diagnosis, yrs, median (min-max)	52 (44–71)	63.5 (37–89)	0.028*
FIGO stage, n (%)			
I-II	4 (36.4)	6 (8.6)	0.026^†^
III-IV	7 (63.6)	64 (91.5)	
Mitotic activity, median (min-max)	9 (1–12)	27 (13–65)	< 0.001*
Vascular invasion, n (%)			
No	10 (90.9)	20 (28.6)	< 0.001^†^
Yes	1 (9.1)	50 (71.4)	
Residual tumor, n (%)			
No	7 (63.6)	12 (17.1)	0.019^†^
≤ 2 cm	1 (9.1)	14 (20.2)	
≥ 2 cm	3 (27.3)	44 (62.9)	

*Based on Mann–Whitney *U* test.

^**†**^ Based on *χ*^2^ test.

### Immunohistochemical evaluation

**Figure 1:**
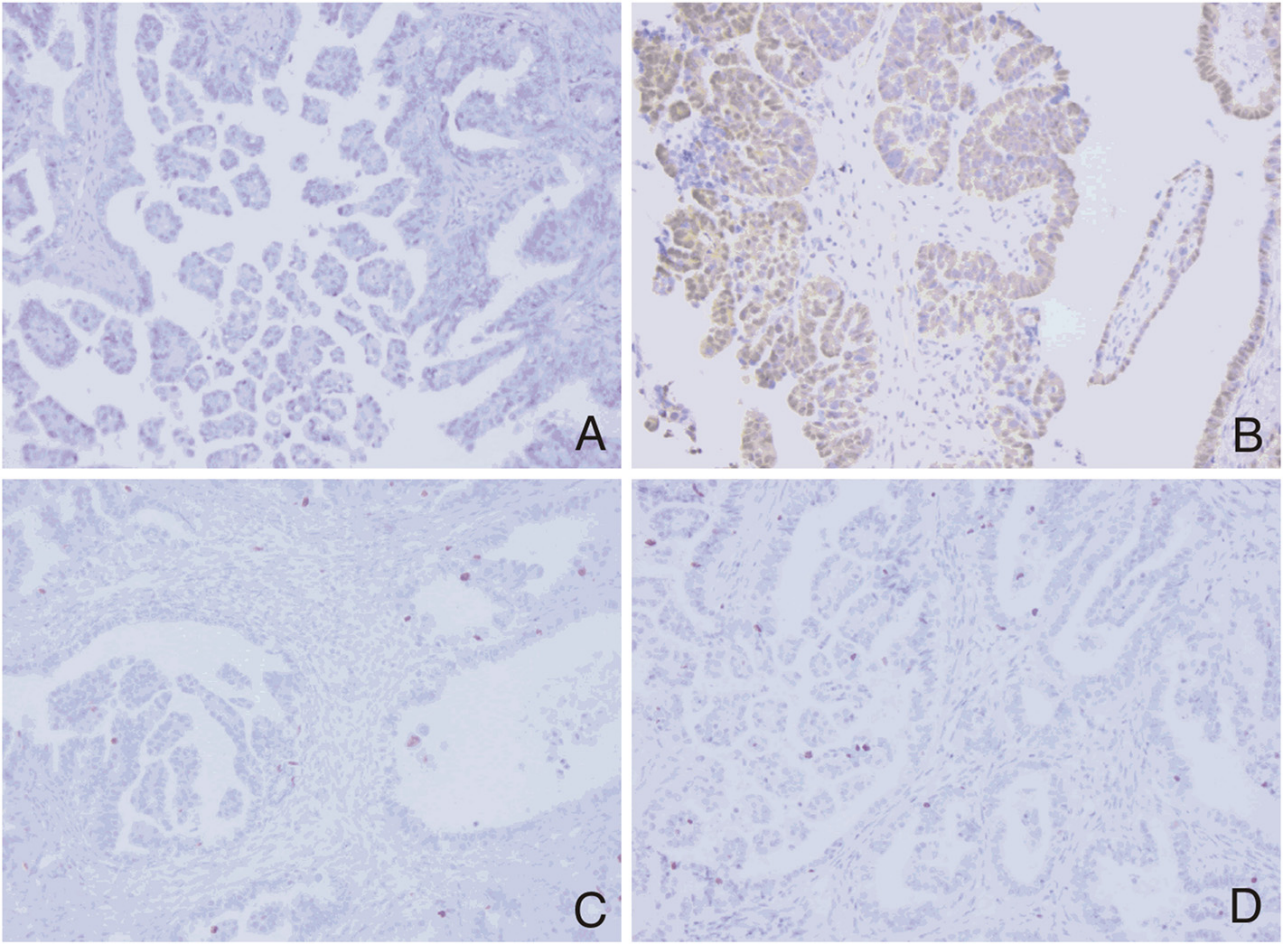
**Representative immunostaining patterns of low-grade OSCs.** Negative p53 immunoexpression (**A**; original magnification, × 200), positive MAPK immunoexpression (**B**; original magnification, × 200), negative topoII alpha immunoexpression (**C**; original magnification, × 200), low Ki67 proliferative activity (**D**; original magnification, × 200).

**Figure 2:**
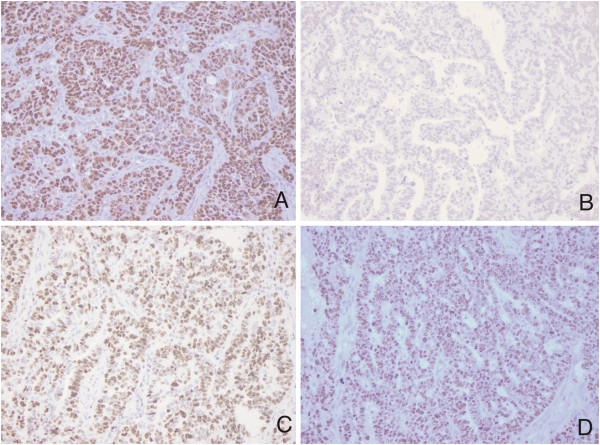
**Representative immunostaining patterns of high-grade OSCs.** Strong p53 immunoexpression (**A**; original magnification, × 200), negative MAPK immunoexpression (**B**; original magnification, × 200), positive topoII alpha immunoexpression (**C**; original magnification, × 200), high Ki67 proliferative activity (**D**; original magnification, × 200).

All of the samples in the low-grade group (100%) exhibited p53 nuclear staining lower than 10% (Figure 1A). In the high-grade group, 85.7% of cases showed strong positive nuclear expression of p53 protein (Figure 2A), while 14.3% of cases showed less than 10% positive nuclei. The observed difference in the p53 protein expression between these two categories was statistically significant (*P* < 0.001).

The difference in expression of MAPK between low- and high-grade group was also significant (χ^2^ = 9.0, *P* = 0.003). MAPK positive staining was detected in 63.6% of low-grade (Figure 1B) as opposed to 17.1% of high-grade carcinomas. The high-grade group is represented with 82.9% of MAPK negative carcinomas (Figure 2B). Ten out of 70 (14.3%) high-grade samples showed simultaneous p53 and MAPK immunoexpression.

There was a significantly higher topoII alpha expression in the high-grade group (Figure 2C) compared to the low-grade group (χ^2^ = 11.2, *P* = 0.001) (Figure 1C). 18.6% of the high-grade carcinomas exhibited less than 10% of positive nuclei.

Significant difference was also observed in the expression of Ki67 between the low- and the high-grade group (z = 4.4, *P* < 0.001). In the low-grade group median was 19 (range, 7–54) as opposed to the high-grade group in which median was 56.5 (range, 18–98) (Figure 1D and Figure 2D).

The results of immunohistochemical staining are shown in Table 2. Representative immunostaining patterns are summarized in Figure 1A-D for low-grade, and Figure 2A-D for high-grade OSCs.

**Table 2:** Immunohistochemical staining results of p53, MAPK, topoIIα and Ki67 expression in OSCs

	**Low-grade**	**High-grade**	***P***
	**(n = 11)**	**(n = 70)**	
p53, n (%)			
negative	11 (100)	10 (14.3)	< 0.001^†^
positive	0 (0)	60 (85.7)	
MAPK, n (%)			
negative	4 (36.4)	58 (82.9)	0.003^†^
positive	7 (63.6)	12 (17.1)	
topoII alpha, n (%)			
negative	7 (63.6)	13 (18.6)	0.001^†^
positive	4 (36.4)	57 (81.4)	
Ki67, median (min-max)	19 (7–54)	56.5 (18–98)	< 0.001^*^

^†^Based on *χ*^2^ test.

*Based on Mann–Whitney *U* test.

### Molecular analysis

KRAS mutation was found in 54.5% of low-grade and 13.8% of high-grade OSCs. The frequency of KRAS mutation was significantly higher in low-grade as compared to high-grade group (*χ*^2^ = 7.4, *P* = 0.006). None of the samples had BRAF mutation. We identified seven (11.7%) high-grade samples that showed both KRAS mutation and p53 immunopositivity.

Furthermore, we compared the findings of KRAS mutational analysis with active MAPK immunoreactivity. As shown in Table 3, the relationship between immunoreactivity and KRAS status is not statistically strong enough to use immunoreactivity to reliably detect KRAS mutation. We observed that 5/6 (83%) of low-grade and 1/8 (12.5%) of high-grade MAPK immunopositive carcinomas contained KRAS mutation. Also, 2/5 (40%) of low-grade and 11/54 (20.4%) of high-grade carcinomas, with wild-type KRAS, showed MAPK positivity. Therefore, MAPK immunopositivity has only limited value in predicting KRAS mutations, with a sensitivity of 0.43, a specificity of 0.78, a positive predictive value of 0.32, and a negative predictive value of 0.85.

**Table 3:** Correlation of MAPK immunoreactivity and mutational status of KRAS in low- and high-grade OSCs

	**Low-grade**	**High-grade**
	**(No. of cases)**	**(No. of cases)**
KRAS mutation		
MAPK +	5	1
MAPK –	1	7
Wild KRAS		
MAPK +	2	11
MAPK –	3	43
Total cases	11	62

## Discussion

Currently, low-grade and high-grade serous carcinomas are thought to represent two distinct pathways of ovarian carcinogenesis, rather than opposite ends of severity along a single trajectory of tumor progression. Recent studies have convincingly demonstrated that morphological differences between these tumors are a manifestation of their underlying biological and genetic disparity. Briefly, low-grade carcinomas evolve along type I pathway and represent relatively indolent neoplasms that arise in a stepwise fashion from well-characterized precursor lesions. High-grade carcinomas are aggressive, genetically unstable neoplasms that arise through type II pathway. However, it remains an open issue whether some high-grade serous carcinomas arise from low-grade serous carcinomas that follow type I pathway [3,4,21].

The proposed dualistic model has important implications for early detection and targeted treatment. Current screening approaches, namely pelvic examinations, CA 125 levels and transvaginal ultrasound are reasonable for low-grade carcinomas, but are not likely to be sufficiently beneficial for high-grade carcinomas. Although the management of these two groups is currently identical, the growing body of evidence suggests that low-grade serous carcinomas are not as responsive as high-grade serous carcinomas to conventional chemotherapy with platinum and taxane agents [22-24].

A better understanding of the molecular pathogenesis of low-grade serous carcinomas would lead to rational evaluation of new targeted agents for the treatment of this disease. Reports point towards a high frequency of KRAS and BRAF mutations in low-grade OSCs, making this pathway an attractive therapeutic target by interfering with its downstream effectors [25,26]. The preliminary promising results of a phase II clinical trial evaluating AZD6244 (selumetenib), an inhibitor of MEK-1/2, have been reported [27].

We report our findings of the immunohistochemical expression of p53, MAPK, topoII alpha and Ki67, and molecular analysis for KRAS and BRAF mutations in the OSCs.

p53 is a tumor suppressor gene located on the short arm of chromosome 17, involved in regulation of cell growth [28]. Despite compelling evidence for the central role of the p53 pathway in human neoplasia, the assessment of p53 status in clinical samples remains unanswered, with confusing and often contradictory literature reports. Methodological differences in the interpretation of the staining results in different studies further contribute to the confusion [18,28-30]. While the correlation between p53 mutational status and immunohistochemical expression is suboptimal, differences in the immunoexpression of p53 in low- and high-grade carcinomas can be diagnostically useful.

There have been a few studies investigating the p53 immunoreactivity in low- and high-grade OSCs [28,31]. In these studies, the extent of immunoexpression was significantly different between low- and high-grade carcinomas (16.7% vs. 53.6%, and 18% vs. 64% of cases exhibited strong staining intensity). Our study confirms significantly higher p53 immunoexpression in the high-grade group (85.7% high-grade carcinomas showed >10% positive cells). In a study by Mishra et al. [31] 22.2% of low-grade samples scored as 0 and 1+ (negative or <10% positive cells). Unlike Mishra’s study, none of our low-grade samples showed more than 10% of p53 immunopositive nuclei.

There is no definitive proof that all low-grade OSCs arise in a stepwise fashion from well-characterized precursor lesions and it is possible that some do not. Likewise, on rare occasions, a low-grade OSC may transform into a high-grade neoplasm [32]. Several studies have shown that, in rare cases, low- and high-grade serous tumors do coexist and/or high-grade serous carcinomas share similar gene expression profile as low-grade carcinomas [14,33].

Therefore, we upgraded the basic morphology and p53 immunoexpression with added MAPK, topo II alpha and Ki67 analysis.

MAPK is a downstream target of the RAS, RAF and MAP/ERK kinases, and is crucial for transduction of growth signals from several key growth factors, cytokines and proto-oncogenes. Mutations (including KRAS and BRAF) or overexpression of upstream components in signal transduction cascades, lead to constitutive activation of MAPK pathway [19]. Because of the frequent KRAS or BRAF mutations in serous tumors that follow type I pathway [3], we examined whether there would be a differential immunoexpression of activated MAPK in our low- and high-grade group.

Nucleocytoplasmic distribution of MAPK is a pivotal point in regulation of its downstream targets. Dual phosphorylation of MAPK on tyrosine and threonine occurs in the cytoplasm. Activated MAPK must translocate into the nucleus to phosphorylate nuclear targets. Active form freely diffuses as a monomer through nuclear pores, homodimerizes and enters the nucleus via a carrier-free/nuclear pore-independent mechanism or interacts with the nuclear pore complex for entry. The nucleus has been proposed to act as an “anchoring and inactivating center” were signal must be terminated by dephosphorylation [34]. We found nuclear and cytoplasmic MAPK in almost all positive samples, which is consistent with previous reports [19,35]. We did not find any difference in localization of positive staining between low- and high-grade group.

In the present study we stated that the immunoexpression of activated MAPK was significantly higher in low-grade as compared to high-grade serous carcinomas. Although the literature on MAPK immunoexpression in serous ovarian tumors is quite limited, our results support findings reported by Hsu et al. [19].

We compared the findings of KRAS mutational analysis with active MAPK immunoreactivity. In this study, frequency of KRAS mutation was significantly higher in low-grade as compared to the high-grade group. Interestingly, none of our OSC samples had BRAF mutation. Similar findings were reported by Wong et al. [36], who detected BRAF mutation in only 2%, and KRAS mutation in 19% of low-grade OSCs. In contrast to our study, they did not detect KRAS or BRAF mutations in their high-grade group. We detected positive MAPK immunoexpression in some low- and high-grade samples with wild-type KRAS, suggesting that activation of MAPK pathway is not ultimately related to KRAS or BRAF mutations.

Seven morphologically high-grade samples (11.7%) showed KRAS mutation, characteristic for type I pathway and p53 immunopositivity, hallmark of type II pathway. However, due to the low number of cases, we refrain from giving a definitive answer to open issues and urge further investigation.

According to our results, unlike the ones of Hsu et al. [19], MAPK immunostaining was not sufficiently sensitive, nor specific, to precisely predict the KRAS mutational status of the tumor. However, MAPK immunostaining appears to be quite reliable in ruling out a KRAS mutation if the staining is negative.

Immunohistochemical expression of topoII alpha in ovarian carcinomas has been demonstrated in several studies, but the results of these studies are difficult to compare because the methodology and criteria for evaluation varied greatly [8,9,37,38]. According to studies on OSCs performed by Brustmann [8,38], the topoII alpha labeling index (LI) increased with mitotic activity (*P* < 0.0004), tumor grade (*P* = 0.0303), FIGO stage (*P* = 0.0076) and indicates poor prognosis (*P* = 0.0182). To the best of our knowledge, no study compared different topoII alpha immunoexpression with regard to proposed dualistic model of ovarian serous carcinogenesis. Based on our results, we report a significantly higher topoII alpha expression in the high-grade group compared to the low-grade group (*P* = 0.001).

As expected, we identified a significant difference between Ki67 immunoexpression in the low-grade and the high-grade group. The results of our study are in broad agreement with previous studies by O’Neill et al. [28] and Mishra et al. [31]. Both groups have shown a lower Ki67 proliferation index in low-grade compared to high-grade OSCs.

The distinction between low- and high-grade serous carcinoma may occasionally be a differential diagnostic problem. Some high-grade serous carcinomas have been shown to mimic low-grade serous carcinomas architecturally. Many of these carcinomas have grade 2 nuclear atypia [21]. Our results indicate that morphologically problematic serous carcinomas with markedly elevated Ki67 proliferation index and positive topoII alpha immunoexpression, are more likely to follow the type II pathway and these markers could be a useful additional tool in distinguishing the low- and high-grade groups of OSCs, along with nuclear atypia and mitotic count.

The findings of our study mostly support the proposed dualistic model of ovarian carcinogenesis. However, morphological examination combined with immunohistochemistry and molecular analyses reveal rare intersections between type I and type II tumorigenic pathway.

## Conclusions

Although this study is limited by its humble number of low-grade samples, our data fit the proposed dualistic pathway of ovarian carcinogenesis. We found statistically significant differences in the immunohistochemical expression of p53, MAPK, topo II alpha and Ki67 between low- and high-grade ovarian cancers along with differences in KRAS mutational status. Immunohistochemical staining for MAPK was not sufficiently sensitive, nor specific, to precisely predict the KRAS mutational status of the tumor. However, it appears to be quite reliable in ruling out a KRAS mutation if the staining is negative. Also, mutational analysis for KRAS and BRAF discloses some possible interactions between type I and type II pathway and could be useful in detection of small proportion of high-grade carcinomas arising through type I pathway, with possible diverse clinical behavior and specific therapy requirements. Those patients might be considered for Ras-Raf-MEK-MAPK-targeting therapies on the basis of molecular profiling data.

## Consent

Written informed consent was obtained from the patient for publication of this report and any accompanying images.

## Abbreviations

OSCs: Ovarian serous carcinomas; MAPK: Mitogen-activated protein kinase; TopoII alpha: Topoisomerase II alpha; HPFs: High power fields.

## Competing interests

The authors declare that no competing interests exist.

## Authors’ contributions

DS and ST contributed to the conception and design of the study, preparation of final manuscript and carried out the histopathological re-evaluation and immunohistochemical evaluation. IDH carried out the molecular analysis. VC drafted the manuscript and carried out the statistical analysis. AC, IM and BPM collected clinical data and drafted the manuscript. DG participated in the evaluation of the immunohistochemistry, drafted and edited the manuscript. All authors read and approved the final manuscript.
